# Patients’ perceptions of male and female nursing interns in Guangdong and Guangxi, Southern China: The interplay of gender stereotypes, patient satisfaction, and willingness to receive care

**DOI:** 10.1097/MD.0000000000050442

**Published:** 2026-08-28

**Authors:** Jun-Juan Liao, Ming Zhou, Xiao-Lan Zeng, Xing-Xing Su, Xiao-Qin Qiu

**Affiliations:** a Department of Urology, Guangxi Hospital Division of The First Affiliated Hospital, Sun Yat-sen University, Nanning, Guangxi Zhuang Autonomous Region, PR China; b Department of Urology, The First Affiliated Hospital of Sun Yat-sen University, Guangzhou, Guangdong Province, PR China; c Department of Urology, The People’s Hospital of Guangxi Zhuang Autonomous Region, Nanning, Guangxi Zhuang Autonomous Region, PR China.

**Keywords:** China, cultural diversity, female nursing interns, gender stereotypes, Guangdong, Guangxi, male nursing interns, patient satisfaction, urban–rural disparity

## Abstract

Nursing remains female-dominated globally, with gender stereotypes hindering workforce diversity. Male nurses account for 2% to 5% of China’s nursing workforce, and Guangdong/Guangxi lack studies on patients’ perceptions of male nursing interns. This study aims to explore relationships between gender stereotypes, patient satisfaction, and willingness to receive care from male/female nursing interns in Guangdong/Guangxi and identify influencing factors. In this study, a multicenter cross-sectional survey (Mar–Jun 2025) enrolled 460 inpatients from 4 tertiary hospitals (2 each in Guangdong/Guangxi) using a culturally adapted questionnaire. Data were analyzed via descriptive statistics, *t*-tests, Pearson’s correlation, and multiple linear regression. Results showed that patients had moderate-to-high willingness to accept male (*M* = 3.65, SD = 1.08) and female (*M* = 4.10, SD = 0.80) interns, with higher willingness for females (*t* = 8.53, *P* < .001). Patient satisfaction was positively correlated (*r* = 0.4 < 0.001), while “empathy as feminine” stereotype was negatively correlated (*r* = −0.40, *P* < .001) with male intern acceptance. Multiple linear regression identified satisfaction (β = 0.35), the stereotype (β = −0.29), urban–rural background (β = 0.16), and education (β = 0.13) as predictors (32.7% variance explained). Male, urban, higher-educated patients had higher willingness; no ethnic differences existed. In conclusion, high-‑quality care was related to lower gender bias, while empathy stereotypes correlated with poorer acceptance. Urban–rural and educational factors were associated with perceptions. Targeted education, clinical training, and gender‑sensitive policies were linked to better acceptance and workforce diversity.

## 1. Introduction

Nursing is globally characterized by severe gender imbalance, with men accounting for only 5% to 15% of the workforce,^[1]^ a disparity that is more pronounced in China (2–5% male nurses)^[2]^ and even further in Southern China’s Guangdong and Guangxi Zhuang Autonomous Region (1.8–3.2%).^[3,4]^ This skewness stems from deep-rooted cultural norms and gender stereotypes that frame nursing as a feminine profession, tied to stereotypically female traits (empathy, gentleness, nurturing capacity).^[5,6]^ As emerging professionals, male nursing interns face unique barriers: doubts over professional suitability, limited hands-on clinical experience (especially in intimate care), and heightened competency pressure,^[7,8]^ which erode their professional identity and drive high attrition – 35% of male nursing students in Guangdong and Guangxi intend to leave the profession due to gender-based discrimination.^[9]^

Patients’ perceptions and attitudes are pivotal to the clinical training environment for nursing interns. Negative stereotypes may lead patients to refuse care from male interns, depriving them of critical basic and intimate care experience (e.g., wound dressing, catheterization),^[10]^ while positive care experiences can challenge such biases and enhance acceptance.^[11,12]^ Studies in Ghana and Pakistan confirm that high care quality makes patients prioritize competence and interpersonal skills over gender, though gender preferences persist in intimate care.^[13,14]^ These dynamics are highly context-dependent, however, necessitating targeted investigation into the unique cultural, socioeconomic, and demographic characteristics of Guangdong and Guangxi.

With a combined population of over 150 million, Guangdong and Guangxi form a distinct research context marked by cultural diversity, stark urban–rural disparities, and uneven nursing workforce distribution. Guangdong is shaped by Lingnan culture and its traditional gender roles (male external/female domestic dominance),^[15]^ while Guangxi – home to ethnic groups including the Zhuang (31.3% of the population, China’s largest ethnic minority), Yao, and Dong – blends matrilineal traditions (in some Zhuang communities) with Confucian gender norms,^[16]^ creating a continuum of gender role attitudes from conservative rural areas to inclusive urban hubs (Guangzhou, Shenzhen, Nanning). Economically, Guangdong’s Pearl River Delta is a highly urbanized, developed region, while rural Guangxi (e.g., Baise, Hechi) retains strong traditional norms and limited access to diverse healthcare teams.^[17,18]^ Additionally, male nurses in both provinces are concentrated in urban tertiary hospitals, with scarce representation in rural/ethnic minority areas, limiting patient exposure to male caregivers.^[19,20]^

Despite growing attention to nursing gender diversity in China, existing studies on patients’ perceptions of male nursing students have critical limitations: most are single-center studies focused on major cities (e.g., Guangzhou, Shanghai) and fail to capture Guangdong and Guangxi’s cultural and socioeconomic diversity,^[21,22]^ and few have systematically examined the interplay between gender stereotypes, patient satisfaction, and willingness to receive care – three intertwined variables shaping patient attitudes.^[23,24]^ This interplay is particularly complex in Guangdong and Guangxi, where cultural norms, urbanization, and ethnic identity intersect to influence gender role perceptions.

Guided by Social Role Theory^[25]^ and Gender Stereotype Theory,^[26]^ this study addresses these gaps. Social Role Theory posits that gender roles derive from traditional labor divisions (communal roles for women, agentic roles for men), meaning male interns may be perceived as non-normative in nursing – a stereotypically communal profession – especially in conservative settings. Gender Stereotype Theory further links caregiving and emotional sensitivity to femininity and technical competence to men; these stereotypes reduce acceptance of male interns in nursing but can be challenged by positive care experiences.

This study had 4 specific objectives: describe patients’ gender stereotypes, care satisfaction, and willingness to receive care from male/female nursing interns in Guangdong and Guangxi; analyze the influence of sociodemographic factors (gender, age, education, urban–rural background, ethnicity) on these variables; explore correlations between stereotypes, satisfaction, and willingness; and identify key predictors of willingness to accept male interns. The findings provide empirical evidence for optimizing nursing education, improving clinical training environments, and promoting gender diversity in Guangdong and Guangxi’s nursing workforce, with implications for other culturally diverse regions in China.

## 2. Materials and methods

### 2.1. Study design

A quantitative, correlational cross-sectional design was adopted to explore the relationships between gender stereotypes, patient satisfaction, and willingness to receive care from male/female nursing interns. This design was selected for its suitability in examining associations between variables in a large, diverse population at a single time point.^[27]^ Cross-sectional studies are widely used in nursing workforce and patient perception research due to their efficiency in data collection and ability to capture a snapshot of attitudes within specific cultural and socioeconomic contexts.^[28,29]^ Written informed consent was secured from all participating physicians and patients. The Ethics Committee of Guangxi Hospital Division of The First Affiliated Hospital, Sun Yat-sen University approved this project, and the approval number is KY-KJT-2023-031.

### 2.2. Participants

**Figure 1.**
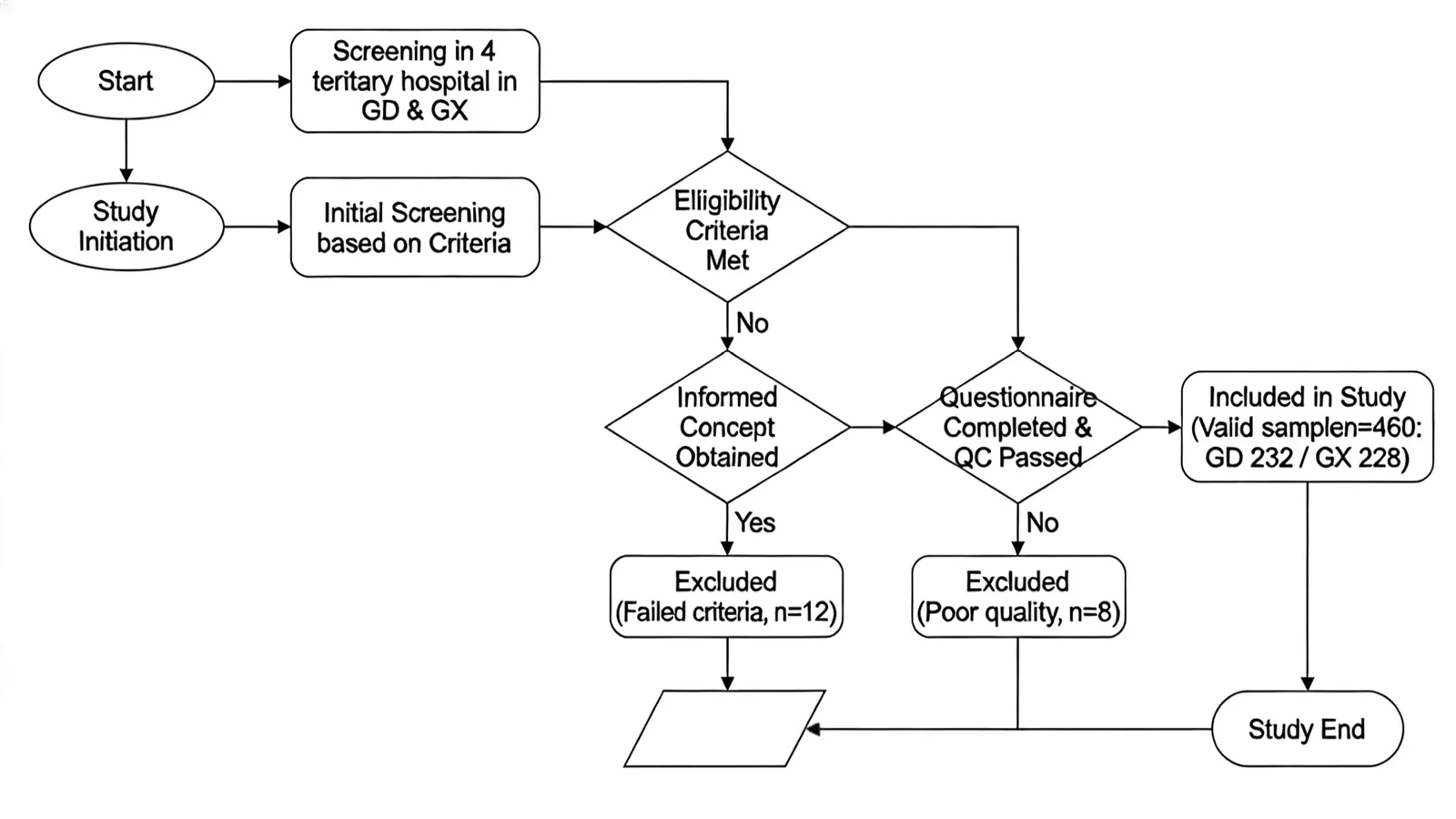
The flow chart of subject recruitment.

**Figure 2.**
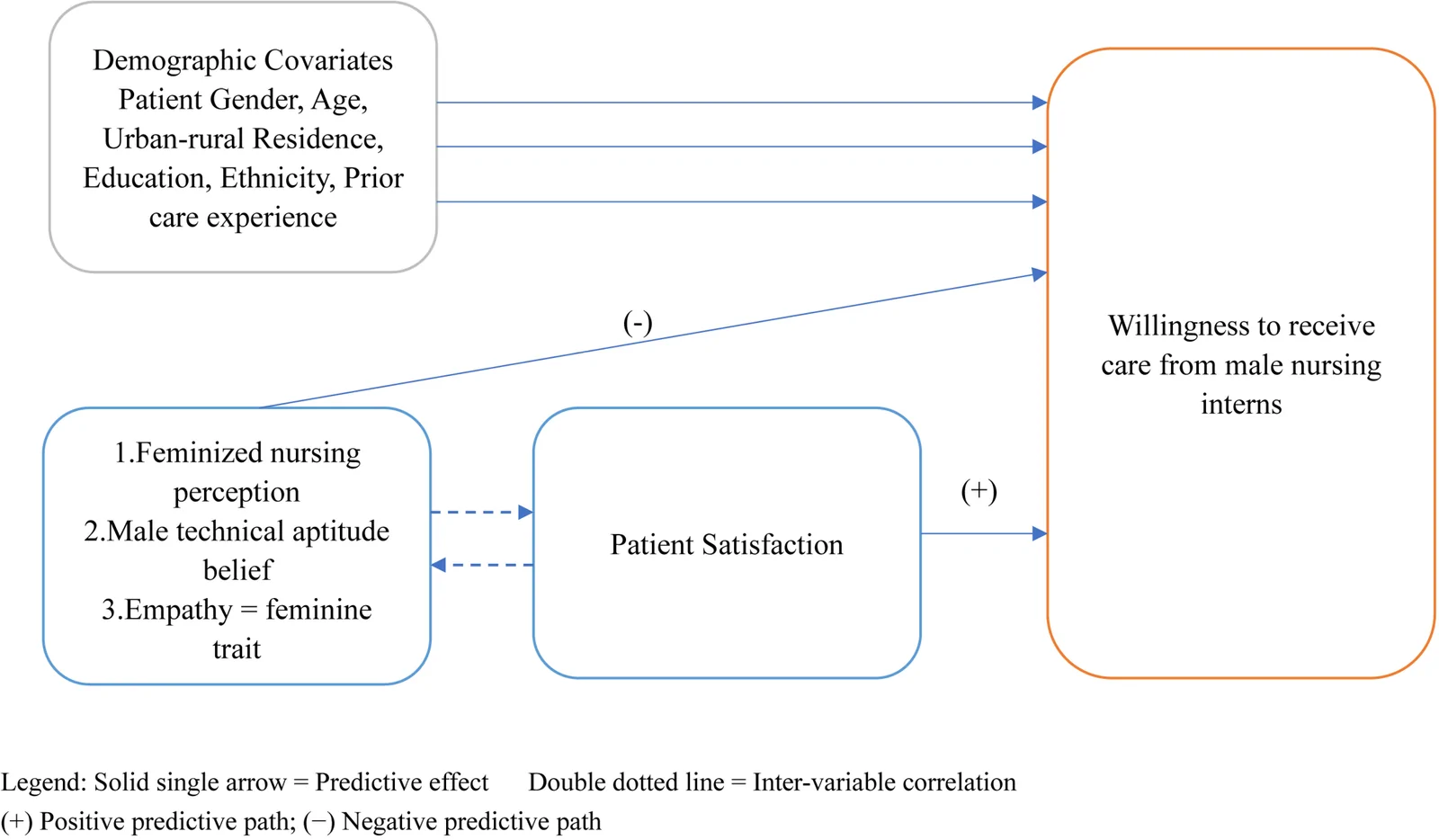
Conceptual theoretical model theoretical basis: social role theory & gender stereotype theory.

The flow chart of subject recruitment is shown in Figure 1. The overall research framework is shown in Figure 2.

#### 2.2.1. Setting

A stratified multicenter sampling strategy was used to recruit participants from 4 tertiary hospitals across Guangdong and Guangxi, ensuring representation of diverse cultural, urban–rural, and ethnic contexts:

#### 2.2.2. Guangdong Province

The First Affiliated Hospital, Sun Yat-sen University. Chaozhou Central Hospital.

#### 2.2.3. Guangxi Zhuang Autonomous Region

The People’s Hospital of Guangxi Zhuang Autonomous Region. Guangxi Hospital Division of The First Affiliated Hospital, Sun Yat-sen University.

### 2.3. Inclusion and exclusion criteria

#### 2.3.1. Inclusion criteria

Age ≥ 18 years (legal adult age in China, ensuring capacity to provide informed consent). Ability to read or understand Mandarin, Cantonese, or Zhuang (the 3 most prevalent languages in the study region), with no communication barriers. Cognitively intact, defined as a Mini-Mental State Examination score ≥ 24^[30]^ – a validated cutoff for intact cognitive function in Chinese populations.^[31]^ Admitted to internal medicine, surgery, or emergency departments (the 3 largest departments in participating hospitals, ensuring exposure to nursing interns). Voluntary participation with written informed consent.

#### 2.3.2. Exclusion criteria

Sedated (receiving continuous intravenous sedation) or in acute pain (numeric rating scale score ≥ 7/10), as these conditions may impair the ability to complete the questionnaire. History of mental illness (schizophrenia, bipolar disorder) or communication disorders (aphasia) documented in medical records. Hospitalization duration < 24 hours or planned discharge within 24 hours (insufficient exposure to nursing interns to form meaningful perceptions). Previous participation in similar studies (to avoid response bias).

### 2.4. Sample size calculation

Sample size was determined using G*Power (3.1.9.7) software for multiple linear regression analysis, the primary statistical method for the study’s key objective (identifying predictors of willingness to accept male interns). The following parameters were set: Significance level (α) = 0.05 (two-tailed). Power (1 − β) = 0.80 (standard for nursing research). Medium effect size (*f*^2^ = 0.15) – consistent with the effect size of predictors including gender stereotypes and patient satisfaction. Number of predictors = 8 (3 stereotype dimensions, patient satisfaction, gender, urban–rural background, education level, ethnicity). The minimum required sample size was 420. Considering a potential 10% nonresponse rate, 460 participants were targeted for recruitment.

### 2.5. Measures

The questionnaire (36 items) was developed through a rigorous “translation-back translation-cultural adaptation-pilot testing” process to ensure linguistic and cultural appropriateness for Southern China. It was available in 4 languages (Mandarin, Cantonese, Minnan, Zhuang) and divided into 4 sections.

#### 2.5.1. Sociodemographic and clinical characteristics (8 items)

Included age (18–30, 31–50, 51–70, >70 years), gender, education level (primary school or below, junior high, senior high/vocational, college or above), urban/rural background, ethnicity (Han, ethnic minority), religious belief (yes/no), hospitalization department (internal medicine, surgery, emergency, others), and history of receiving care from male/female nursing students (yes/no).

#### 2.5.2. Patient Satisfaction Scale (5 items)

Adapted from the Chinese version of the Patient Satisfaction with Nursing Care Quality Questionnaire,^[32]^ which has been validated in Chinese clinical settings. This scale assesses patients’ satisfaction with 5 dimensions: professional skills, communication attitude, care timeliness, humanistic care, and overall care of nursing students. Responses were rated on a 5-point Likert scale (1 = Not satisfied at all, 5 = Very satisfied). In this study, the Cronbach’s α coefficient was 0.86, indicating high internal consistency.

### 2.6. Gender Stereotype Scale (15 items)

Adapted from the Attitude toward Men in Nursing Questionnaire,^[33]^ a validated scale with strong psychometric properties. Cultural adaptation was conducted to align with Guangdong and Guangxi’s cultural context:

#### 2.6.1. Translation and back-translation

The original English scale was translated into Mandarin by 2 bilingual nursing researchers (fluent in English and Mandarin, with expertise in gender studies). A third independent bilingual researcher (not involved in the initial translation) back-translated the Mandarin version into English. Discrepancies were resolved through a consensus meeting with the research team.

1. Cultural adjustment replaced “religious norms” with “traditional Lingnan/Zhuang cultural norms” – since religion has minimal influence on gender roles in the study region, while cultural norms are dominant. Added 2 items reflecting local gender role perceptions: “Male nursing interns should not provide intimate care to female patients” and “Zhuang women are more suitable for caregiving than Zhuang men.” Modified item wording to match local dialectical expressions.
2. Content validity: 3 experts (a nursing professor specializing in gender research, a sociologist with expertise in Southern Chinese culture, and a clinical nurse manager with 15 years of experience in Guangxi) evaluated the scale for relevance, clarity, and cultural appropriateness. The content validity index (CVI) was 0.92 (item-level CVI range: 0.83–1.00), indicating excellent content validity.
3. The final scale retains 3 dimensions consistent with the original AMnQ: perceptions of nursing as a feminized and low-status profession (5 items); belief that male nurses are more suited to technical or physically demanding tasks (5 items); association of empathy and caregiving with femininity (5 items).

Responses were rated on a 5-point Likert scale (1=“Strongly disagree” to 5=“Strongly agree”). In the current study, the Cronbach’s α coefficient was 0.83 for the total scale, and 0.77, 0.80, and 0.82 for the 3 subscales, respectively – indicating good internal consistency.

#### 2.6.2. Willingness to Receive Care Scale (8 items)

Developed based on Social Role Theory and Gender Stereotype Theory, with reference to Ben Natan et al’s scale and localized adjustments for Guangdong and Guangxi. The scale includes 2 parallel subscales:

1. Willingness to accept male nursing interns;
2. Willingness to accept female nursing interns.

Responses were rated on a 5-point Likert scale (1=“Strongly disagree” to 5=“Strongly agree”). Pilot testing (n = 30 patients, including 10 Zhuang patients) confirmed the scale’s clarity and relevance (item-level CVI = 0.95). In the current study, the Cronbach’s α coefficients were 0.86 for the male intern subscale and 0.82 for the female intern subscale – indicating high internal consistency.

#### 2.6.3. Pilot testing

A pilot study was conducted among 30 eligible patients (5 from each participating hospital) between January and February 2025. Participants completed the questionnaire and provided feedback on item clarity, cultural relevance, and completion time. Key revisions included: Simplifying complex wording. Adding examples for ambiguous items. Reducing completion time from 20 to 25 minutes to 15 to 20 minutes by removing redundant wording. No major structural changes were needed, confirming the questionnaire’s feasibility and appropriateness for the target population.

### 2.7. Data collection

Data collection was conducted between March and June 2025, a period with stable hospital admission volumes (avoiding peak seasons such as Spring Festival or flu outbreaks). Patient identification: eligible patients were identified 24 to 72 hours after admission. This time window was selected to ensure patients were stable (post-admission acute phase passed) and had sufficient exposure to nursing interns (most interns rotate through wards within 24 hours of patient admission). Informed consent: research assistants approached patients face-to-face, explained the study purpose, procedures, anonymity, and right to withdraw at any time without negative consequences. Written informed consent was obtained, and a copy was provided to the patient. Questionnaire administration: the appropriate language version (Mandarin/Cantonese/Zhuang) was distributed. Patients completed the paper-based questionnaire independently; for those with low literacy (self-reported or identified by research assistants), the questionnaire was read aloud without inducing responses, and patients’ answers were recorded. Quality control: completed questionnaires were reviewed on-site by research assistants to check for missing data. Partial responses (≥3 missing items) were excluded; questionnaires with 1 to 2 missing items had missing values imputed using the mean score of the corresponding subscale. Data encoding: each questionnaire was assigned a unique identifier to ensure anonymity. No personal identifying information was collected. A total of 480 questionnaires were distributed, and 460 were completed and returned, yielding a response rate of 95.8%. Reasons for nonparticipation included fatigue (n = 12), clinical deterioration (n = 5), and early discharge (n = 3).

### 2.8. Data analysis

All data analyses were performed with IBM SPSS Statistics software (Version 29). Descriptive statistical measures, encompassing frequency distributions, arithmetic means, and standard deviations (SDs), were employed to delineate the demographic profiles of the study sample and the distribution characteristics of all focal study variables. For inferential statistical assessments, Pearson’s correlation coefficients were first calculated to quantify the linear associations among continuous variables, followed by independent-samples *t*-tests to compare the mean differences of key indicators across distinct study groups.

Variable selection for regression modeling was guided by established theoretical foundations and strictly aligned with the a priori conceptual framework of the present study. Prior to implementing multiple linear regression analyses aimed at identifying predictors of participants’ willingness to accept care provided by male nursing students, a rigorous verification of critical statistical assumptions was completed. The normality of regression residuals was evaluated using quantile–quantile (Q–Q) plots combined with the Shapiro–Wilk test. Homoscedasticity was assessed by constructing scatterplots that plotted standardized residuals against predicted values, to confirm the homogeneity of variance across the range of predicted outcomes. Multicollinearity among candidate predictor variables was examined via the calculation of variance inflation factors; all variance inflation factor values were below the threshold of 2.0, indicating the absence of problematic multicollinearity. No substantial violations of the core statistical assumptions were detected throughout these checks. Statistical significance was defined as a 2-tailed *P*-value of less than .05 for all inferential tests.

Categorical variables included in the regression model were treated as dummy variables. Gender (male = 1, female = 0), urban‑rural background (urban = 1, rural = 0), prior care experience (yes = 1, no = 0), and ethnicity (Han = 1, Zhuang = 0) were dummy coded to avoid multicollinearity. Univariate outliers were identified using *Z*‑scores with a threshold of |*Z*| > 3.0. Multivariate outliers were detected using Mahalanobis distance (*P* < .001). No extreme outliers were found that would bias the results. The proportion of missing data was less than 5% across all variables. Missing values were handled using listwise deletion, which is appropriate and reliable under a low missing rate.

## 3. Results

### 3.1. Sociodemographic characteristics

Table 1 presents the sample characteristics (N = 460). The mean age was 44.8 years (SD = 18.1, range = 18-91). Most participants were female (53.3%, n = 245), Han ethnicity (78.9%, n = 363), urban residents (59.1%, n = 272), and had senior high/vocational education or above (60.9%, n = 280). Over half (57.0%, n = 262) had received care from male interns, and 88.7% (n = 408) from female interns. Ethnic minorities included Zhuang (15.9%, n = 73), Yao (2.4%, n = 11), and others (2.8%, n = 13). According to the territorial division of the participating hospitals, the sample was divided into Guangdong Province (n = 232) and Guangxi Zhuang Autonomous Region (n = 228), with the basic demographic characteristics of the 2 subgroups balanced and comparable.

**Table 1:** Sociodemographic and clinical characteristics of the study sample (N = 460).

Variable	n (%)	*M* (SD)	Range
Age (yr)	–	44.8 (18.1)	18–91
Gender			
Male	215 (46.7)	–	–
Female	245 (53.3)	–	–
Education level			
Primary school or below	90 (19.6)	–	–
Junior high	91 (19.8)	–	–
Senior high/vocational	156 (33.9)	–	–
College or above	123 (26.7)	–	–
Urban/rural background			
Urban	272 (59.1)	–	–
Rural	188 (40.9)	–	–
Ethnicity			
Han	363 (78.9)	–	–
Zhuang	73 (15.9)	–	–
Other ethnic minorities	24 (5.2)	–	–
Hospitalization department			
Internal medicine	201 (43.7)	–	–
Surgery	189 (41.1)	–	–
Emergency	40 (8.7)	–	–
Others	30 (6.5)	–	–
Received care from male interns			
Yes	262 (57.0)	–	–
No	198 (43.0)	–	–

### 3.2. Descriptive statistics of main variables

Table 2 shows descriptive statistics. Willingness to accept female interns (*M* = 4.10, SD = 0.80) was significantly higher than that for male interns (*M* = 3.65, SD = 1.08; *t* = 8.53, *P* < .001). Patient satisfaction was high (*M* = 3.93, SD = 0.83), with 61.7% (n = 284) “satisfied” or “very satisfied” with male interns. Stereotypical perceptions were moderate to low: “association of empathy with femininity” (*M* = 2.83, SD = 1.05) scored highest, followed by “perceptions of nursing as feminized” (*M* = 2.47, SD = 0.96), and “belief in male technical suitability” (*M* = 2.25, SD = 0.90).

**Table 2:** Descriptive statistics for main study variables (N = 460).

Variable	*M* (SD)
Willingness to accept male interns	3.65 (1.08)
Willingness to accept female interns	4.10 (0.80)
Patient satisfaction	3.93 (0.83)
Gender stereotypes (total scale)	2.51 (0.88)
Perceptions of nursing as feminized	2.47 (0.96)
Belief in male technical suitability	2.25 (0.90)
Association of empathy with femininity	2.83 (1.05)

All variables rated on a 5-point Likert scale (1 = Strongly disagree/Not satisfied, 5 = Strongly agree/Very satisfied).

### 3.3. Inter-provincial comparison of core variables

Independent samples *t*-tests were used to compare the differences in core research variables between Guangdong and Guangxi subgroups (Table 3). The results showed that there were statistically significant differences in the willingness to accept male nursing interns, overall patient satisfaction, and the stereotype dimension of “association of empathy with femininity” between the 2 provinces/autonomous regions (all *P* < .05), while there were no significant differences in the willingness to accept female nursing interns, “perceptions of nursing as feminized and low-status profession” and “belief that male nurses are more suited to technical or physically demanding tasks” (all *P* > .05). Specifically, the scores of willingness to accept male nursing interns (*M* = 3.76, SD = 1.02) and overall patient satisfaction (*M* = 4.02, SD = 0.78) in Guangdong Province were significantly higher than those in Guangxi Zhuang Autonomous Region (*M* = 3.53, SD = 1.12; *M* = 3.83, SD = 0.86). In contrast, the score for “association of empathy with femininity” in Guangxi Zhuang Autonomous Region (*M* = 2.95, SD = 1.07) was significantly higher than that in Guangdong Province (*M* = 2.71, SD = 1.01). We constructed a dedicated new table, Table 4: Psychometric Properties of the Four Study Questionnaires (N = 460). This table systematically summarizes all psychometric indicators of the 4 measurement tools used in this research for quick reference by readers.

**Table 3:** Inter-provincial comparison of core research variables (N = 460).

Research variables	Guangdong Province (n = 232)	Guangxi Zhuang Autonomous Region (n = 228)	Mean difference (GD-GX)	95% CI for mean difference	*t* value	*P* value
Willingness to accept male nursing interns	3.76 ± 1.02 [3.63, 3.89]	3.53 ± 1.12 [3.38, 3.68]	0.23	[0.02, 0.44]	2.15	.032*
Willingness to accept female nursing interns	4.15 ± 0.75 [4.05, 4.25]	4.05 ± 0.84 [3.94, 4.16]	0.1	[-0.05, 0.25]	1.28	.201
Overall patient satisfaction	4.02 ± 0.78 [3.92, 4.12]	3.83 ± 0.86 [3.72, 3.94]	0.19	[0.03, 0.35]	2.37	.018*
Association of empathy with femininity	2.71 ± 1.01 [2.58, 2.84]	2.95 ± 1.07 [2.81, 3.09]	−0.24	[−0.45, −0.03]	-2.29	.022*
Perceptions of nursing as feminized and low-status profession	2.39 ± 0.93 [2.27, 2.51]	2.55 ± 0.98 [2.42, 2.68]	−0.16	[−0.34, 0.02]	-1.72	.086
Belief that male nurses are more suited to technical or physically demanding tasks	2.18 ± 0.87 [2.07, 2.29]	2.32 ± 0.92 [2.20, 2.44]	−0.14	[−0.31, 0.03]	-1.61	.108

All variables measured on a 5-point Likert scale (1 = Strongly Disagree/Not Satisfied; 5 = Strongly Agree/Very Satisfied). Equal variances assumed via Levene’s test for all comparisons. Supplementary descriptive statistics (standard error of the mean, SEM) for each group can be calculated as SD/√n upon reader request. All complete group comparison statistics are fully displayed without truncation.

GD = Guangdong Province; GX = Guangxi Zhuang Autonomous Region, SD = standard deviation.

* *P* < .05, indicating a statistically significant difference between the 2 groups.

**Table 4:** Psychometric properties of the Four Study Questionnaires (N = 460).

Questionnaire scale	Number of items	Subdimensions	Cronbach’s α (Subscale)	Cronbach’s α (total scale)	Content validity index (CVI)	Source & cultural adaptation
Sociodemographic questionnaire	8	Single dimension	–	—	N/A	Self-designed, demographic inventory
Patient satisfaction scale	5	Single dimension	–	0.86	0.91	Adapted from Chinese nursing satisfaction scale; minor wording localization
Gender stereotype scale	15	1. Nursing as feminized low-status profession. 2. Male suitability for technical tasks3. Empathy linked to femininity	0.770.800.82	0.83	0.92	AMnQ original scale; translation-back translation + Lingnan/Zhuang cultural revision
Willingness to receive care scale	8	1. Willingness to accept male interns. 2. Willingness to accept female interns	0.860.82	0.84	0.95	Developed based on Social Role Theory; localized for southern China clinical context

Cronbach’s α ≥ 0.70 indicates acceptable internal consistency; overall scale CVI ≥ 0.90 represents excellent content validity. All scales adopt 5-point Likert response format.

### 3.4. Group differences in willingness

Regarding the factors influencing patients’ willingness to accept care from male interns, significant differences were observed across gender, urban/rural background, educational level, and care experience, while no significant difference was found in ethnicity. Specifically, male patients (*M* = 3.90, SD = 0.93) were more willing to accept male interns than female patients (*M* = 3.41, SD = 1.09; *t* = 4.79, *P* < .001); urban residents (*M* = 3.83, SD = 0.99) exhibited higher willingness than rural residents (*M* = 3.32, SD = 1.14; *t* = 4.45, *P* < .001); college-educated patients (*M* = 3.94, SD = 0.89) showed higher willingness compared with those with primary education (*M* = 3.18, SD = 1.20; *F* = 13.21, *P* < .001); and patients who had received care from male interns (*M* = 3.84, SD = 0.98) reported higher willingness than those who had not (*M* = 3.43, SD = 1.12; *t* = 4.<0.001). In contrast, there was no significant difference in willingness between Han patients (*M* = 3.67, SD = 1.07) and ethnic minority patients (*M* = 3.56, SD = 1.12; *P* = .38).

The stratified analysis by provinces/autonomous regions showed that the influencing trends of urban–rural background and educational level on patients’ willingness to accept male nursing interns were highly consistent in Guangdong and Guangxi. In both regions, urban residents and patients with higher educational levels had significantly higher willingness to accept male nursing interns than rural residents and patients with lower educational levels (all *P* < .05). Meanwhile, no significant differences in all research variables were found between Han and ethnic minority patients in either Guangdong or Guangxi (all *P* > .05), indicating that the influencing effect of ethnic factors was not significant across regions.

We further performed an ethnic subgroup analysis comparing Han and Zhuang participants, the 2 major ethnic groups in this study. Independent samples *t*-tests revealed no significant differences between Han and Zhuang patients in gender stereotypes or willingness to accept male nursing interns (all *P* > .05). Other ethnic groups were not included in the subgroup comparison due to extremely small sample sizes, which may limit the reliability and stability of statistical results.

### 3.5. Correlation analysis

Willingness to accept male interns was positively correlated with patient satisfaction (*r* = 0.43, *P* < .001) and negatively correlated with all 3 gender stereotype dimensions (Table 5). The respective correlation coefficients were *r* = −0.40 (*P* < .001) for “association of empathy with femininity,” *r* = −0.36 (*P* < .001) for “perceptions of nursing as feminized,” and *r* = −0.22 (*P* < .001) for “belief in male technical suitability.” Willingness to accept female interns had a weak positive correlation with satisfaction (*r* = 0.34, *P* < .001) and no significant correlation with gender stereotypes (Table 5).

**Table 5:** Pearson correlations between key variables (N = 460).

Variable	1	2	3	4	5	6
1. Willingness to accept male interns	–	0.39**	0.43**	−0.36**	−0.22**	−0.40**
2. Willingness to accept female interns	0.39**	–	0.34**	−0.07	−0.05	−0.09
3. Patient satisfaction	0.43**	0.34**	–	−0.29**	−0.25**	−0.27**
4. Perceptions of nursing as feminized	−0.36**	−0.07	−0.29**	–	0.52**	0.83**
5. Belief in male technical suitability	−0.22**	−0.05	−0.25**	0.52**	–	0.57**
6. Association of empathy with femininity	−0.40**	−0.09	−0.27**	0.83**	0.57**	–

** *P* < .01.

### 3.6. Predictors of willingness to accept male interns

Multiple linear regression analysis identified 4 significant predictors of patients’ willingness to accept male interns (Table 6), which collectively explained 32.7% of the variance in the outcome variable (*R*^2^ = 0.327, adjusted *R*^2^ = 0.313, *F* = 23.89, *P* < .001). Specifically, patient satisfaction was a positive predictor (β = 0.35, *P* < .001), indicating that higher levels of patient satisfaction were associated with greater willingness to accept male interns. In contrast, the stereotype of associating empathy with femininity was a negative predictor (β = −0.29, *P* < .001), meaning that a stronger adherence to this stereotype predicted lower acceptance. Additionally, urban–rural background (β = 0.16, *P* = .001) and education level (β = 0.13, *P* = .008) were positive predictors, with urban residents and patients with higher educational levels demonstrating greater willingness to accept male interns.

**Table 6:** Predictors of willingness to accept male nursing interns (N = 460).

Predictor	B	SE B	β	*t*	*P*
Patient satisfaction	0.421	0.075	0.350	5.613	<.001
Association of empathy with femininity stereotype	−0.312	0.067	−0.287	−4.652	<.001
Urban/rural background (reference: rural)	0.235	0.076	0.158	3.098	.001
Education level (reference: primary school)	0.192	0.072	0.128	2.664	.008
Patient gender	0.086	0.079	0.041	1.089	.277
Age	−0.002	0.002	−0.034	−0.871	.384
Ethnicity	0.053	0.09	0.021	0.589	.556
Prior experience receiving care from male interns	0.071	0.077	0.033	0.922	.357
Constant	2.107	0.211	–	9.986	<.001

All continuous predictors entered in raw metric form; categorical covariates were dummy coded. Complete model fit indices are provided below the table, including overall *F*-test, *R*^2^, and adjusted *R*^2^. Nonsignificant covariates (*P* > .05) were retained in the model to control for demographic confounding effects as prespecified in the study’s analytical plan.

β = standardized coefficient; B = unstandardized coefficient; SE = standard error.

To examine potential subgroup differences in the predictive effects of core variables and verify interaction effects, hierarchical multiple linear regression analysis was conducted to test the 2 key interaction terms (patient satisfaction × urban–rural background; association of empathy with femininity × urban–rural background). Main effects (patient satisfaction, association of empathy with femininity, urban–rural background, education level) were entered in Step 1, and the 2 interaction terms were added in Step 2. Results showed that no significant interaction effects were detected (all *P* > .05): the interaction term of patient satisfaction × urban–rural background (β = 0.04, *P* = .528) was not statistically significant, and the interaction term of association of empathy with femininity × urban–rural background (β = −0.03, *P* = .615) also showed no statistical significance.

Further stratified multiple linear regression analyses were performed for urban (n = 272) and rural (n = 188) subgroups with the same set of predictors, which confirmed the consistency of core predictive effects across subgroups. For the urban subgroup, patient satisfaction (β = 0.33, *P* < .001) was a significant positive predictor and association of empathy with femininity (β = −0.27, *P* < .001) was a significant negative predictor, with the model explaining 34.2% of the variance (adjusted *R*^2^ = 0.328). For the rural subgroup, patient satisfaction (β = 0.37, *P* < .001) remained a significant positive predictor and association of empathy with femininity (β = −0.31, *P* < .001) was a significant negative predictor, with the model explaining 31.5% of the variance (adjusted *R*^2^ = 0.301). The magnitude of regression coefficients for the core predictors showed no notable differences between urban and rural subgroups, further verifying that the predictive effects of patient satisfaction and the empathy-femininity stereotype on willingness to accept male interns were consistent across urban and rural patient populations, with no significant subgroup differences.

## 4. Discussion

This study explores the interplay of gender stereotypes, patient satisfaction, and willingness to receive care from nursing interns in Guangdong and Guangxi, 2 southern Chinese provinces with distinctive cultural diversity and socioeconomic disparities. The findings reveal nuanced dynamics that align with global research trends while reflecting region-specific characteristics shaped by local cultural norms, urban–rural divides, and nursing workforce distribution.

Patients’ willingness to accept male nursing interns was moderate (*M* = 3.65), significantly lower than that for female interns (*M* = 4.10). This gap is consistent with global evidence^[34,35]^ and more pronounced than in Israel,^[36]^ where male nurse representation (18%) is far higher than in Guangdong and Guangxi (1.8–3.2%).^[37,38]^ Limited patient exposure to male caregivers in the 2 provinces is associated with reinforced stereotypes of nursing as a “female profession.”^[39]^ The urban–rural disparity in acceptance gaps (rural Δ*M* = 0.58 vs urban Δ*M* = 0.42) correlates with the persistence of traditional gender roles in rural communities, where Lingnan and Zhuang cultural norms frame caregiving as women’s work.^[40,41]^ Inter-provincially, Guangdong patients reported higher willingness to accept male interns (*M* = 3.76 ± 1.02) and overall care satisfaction (*M* = 4.02 ± 0.78) than Guangxi patients (willingness *M* = 3.53 ± 1.12; satisfaction *M* = 3.83 ± 0.86). This difference verifies that patient satisfaction is a key positive predictor of willingness to accept male interns and reflects regional heterogeneity in patient perceptions associated with socioeconomic and cultural development gaps between the 2 regions.

Patient satisfaction was the strongest positive predictor of willingness to accept male interns (β = 0.35), consistent with global studies.^[36,42,43]^ This finding highlights that high-quality care is associated with reduced impact of gender stereotypes, even in culturally conservative regions. Patients with high ratings for male interns frequently cited thorough communication, reliable technical skills, and respect for cultural norms as key associated factors, aligning with Social Role Theory^[44]^: male interns who demonstrate competence and meet nursing’s core role expectations (caregiving, empathy) are perceived as less deviant from normative gender roles. For instance, positive care experiences from male interns were reported to alter initial negative attitudes among ethnic minority patients in rural Guangxi. Inter-provincial data further support this association: Guangdong’s higher care satisfaction is linked to a weaker tendency to associate empathy with femininity (*M* = 2.71 ± 1.01 vs Guangxi *M* = 2.95 ± 1.07), suggesting that positive care experiences in more economically developed regions correlate with reduced core stereotypes associated with lower acceptance of male interns.

The stereotype that “empathy and care are feminine traits” was the strongest negative predictor (β = −0.29), mirroring Gender Stereotype Theory.^[36,45]^ This stereotype is deeply entrenched in Guangdong and Guangxi due to cultural norms: Lingnan culture emphasizes “female gentleness” as a core caregiving trait,^[46]^ and Zhuang traditions – even with matrilineal roots in some communities – have adopted Confucian gender roles linking empathy to women.^[47]^ This stereotype is more prominent in Guangxi, a factor associated with the province’s lower patient willingness to accept male interns, and correlates with Guangxi’s higher rural and ethnic minority population share where traditional gender norms persist. In contrast, Guangdong’s higher urbanization and economic development are associated with more open gender concepts. Female patients in particular expressed discomfort with male interns providing intimate care, a concern linked to Confucian modesty norms and more pronounced than in Western secular contexts. Notably, higher care satisfaction correlated with reduced discomfort: 45% of female patients with “very satisfied” ratings for male interns reported willingness to receive future intimate care, compared to only 12% of dissatisfied patients.

Urban–rural background (β = 0.16) and education level (β = 0.13) were significant positive predictors of acceptance, reflecting socioeconomic and cultural divides in the 2 provinces. Urban residents, especially in Guangzhou and Shenzhen, are exposed to diverse social norms, higher male nurse representation in tertiary hospitals, and modern gender equality discourse – factors associated with more inclusive attitudes.^[48]^ Rural areas (e.g., Baise, Chaozhou) retain strong traditional norms and limited access to male nurses, correlates of reinforced stereotypes. Similarly, higher education is associated with recognition of nursing as a gender-neutral profession, while lower education correlates with adherence to cultural myths about gender and care.^[49]^ Stratified analysis showed consistent trends across Guangdong and Guangxi: urban and highly educated patients had significantly higher acceptance of male interns in both regions, indicating that urbanization and education are universal structural factors associated with patient perceptions of male nursing interns in southern China. No significant ethnic differences in willingness were observed between Han and predominantly Zhuang patients, contradicting initial hypotheses about the influence of Zhuang matrilineal heritage but aligning with recent research^[50]^ showing modernization and improved healthcare access correlate with reduced ethnic differences in gender role perceptions. Instead, individual care experiences and satisfaction were more strongly associated with acceptance than ethnic identity, suggesting clinical interactions may override cultural differences – a finding consistent across both provinces in all research variables.

Our findings align with global research while highlighting unique regional challenges in Guangdong and Guangxi. Like Ghana,^[51]^ patients prioritized competence over gender, but Chinese patients more frequently cited modesty as a barrier to male intern care, a factor linked to Confucian “li” (propriety) norms emphasizing gender segregation in intimate contexts.^[52]^ As in Pakistan,^[53]^ respectful and supportive care from male nurses correlated with reduced cultural biases, but Pakistan’s higher male nurse representation (12%) provides more opportunities for positive patient interactions than in Guangdong and Guangxi. Within China, our results extend single-center studies by capturing the cultural and socioeconomic diversity of southern China: Beijing^[54]^ reported moderate male nurse acceptance (*M* = 3.52), similar to our sample, but northern China’s more patriarchal norms may correlate with distinct bias dynamics, while Guangdong and Guangxi’s economic development and cultural openness are associated with softened traditional gender roles, explaining relatively higher acceptance despite ethnic diversity. Shanghai^[55]^ identified urban–rural attitude differences, and our study adds that rural patients in Guangxi who interacted with male interns showed similar willingness to urban patients (*M* = 3.71 vs *M* = 3.83, *P* = .34), a finding linking care experience to reduced urban–rural disparities in acceptance. Compared to Israel,^[36]^ our study identified urban–rural background and education as additional predictors – reflecting China’s larger socioeconomic disparities, in contrast to Israel’s high urbanization (92%) and homogeneous education levels.^[56]^ Israel’s study found no gender differences in patient willingness, while our study showed male patients were more accepting, a difference associated with China’s lower male nurse representation and stronger gender role segregation in care contexts.

This study advances the contextual application of Social Role Theory and Gender Stereotype Theory in Guangdong and Guangxi’s unique cultural and socioeconomic setting. Social Role Theory posits gender roles are shaped by labor divisions, and our findings show these roles are malleable in clinical contexts: male interns who fulfill communal caregiving roles (empathy, communication) correlate with revised normative gender expectations among patients. Gender Stereotype Theory links empathy to femininity, and our data show this link weakens with high patient satisfaction, suggesting stereotypes are not fixed and correlate with positive clinical interactions. Inter-provincial comparisons further enrich theoretical application in China: differences in gender role malleability and stereotype persistence between Guangdong and Guangxi verify that social role and gender stereotype influences on patient perceptions are not only associated with individual clinical experience but also constrained by regional socioeconomic and cultural contexts. Additionally, the study highlights that contextual factors (urban–rural background, education) moderate the stereotype-attitude relationship, reflecting structural inequalities associated with exposure to diverse caregivers and gender norms, and adding a socioeconomic dimension to existing theories. Rural patients’ stronger stereotypes are associated with both cultural norms and structural factors (limited access to male nurses, lower education) that reduce exposure to modern gender discourse, with inter-provincial gaps in urbanization, education, and economic development representing the macro-structural manifestations of these divides in southern China.

### 4.1. Study generalizability

This study’s findings have partial generalizability to other culturally diverse, socioeconomically disparate regions in southern and southwestern China with similar ethnic compositions, urban–rural divides, and low male nurse representation (e.g., Yunnan, Guizhou). The consistent associations of urbanization, education, and care satisfaction with patient acceptance of male interns across Guangdong and Guangxi suggest these factors may be universal in shaping patient perceptions in Chinese contexts with comparable structural characteristics. However, the study’s sampling focused on general care wards in tertiary and secondary hospitals in the 2 provinces, with no inclusion of specialized wards (e.g., obstetrics, gynecology, pediatrics). This specialty-specific sampling limits generalizability to specialized care settings, where gender stereotypes and patient acceptance may differ due to the intimate nature of care and stronger cultural gender norms. Further, the sample overrepresents hospital-based patients, and findings may not extend to community healthcare settings in Guangdong and Guangxi, where patient demographics and care expectations differ.

### 4.2. Study limitations

First, the cross-sectional design prevents causal inference; all observed relationships (e.g., satisfaction and acceptance, stereotypes and willingness) are correlational, and reverse causality cannot be ruled out (e.g., preexisting willingness to accept male interns may correlate with higher care satisfaction). Second, the study employed specialty-specific sampling focused on general wards, excluding specialized care settings, which limits the generalizability of findings to the broader nursing context in Guangdong and Guangxi. Third, data collection relied on self-reported patient questionnaires, which may be subject to social desirability bias – patients may underreport negative attitudes toward male interns to align with social norms of gender equality. Fourth, the study did not measure the frequency and quality of patients' prior interactions with male nurses, a potential confounding variable associated with both stereotypes and acceptance willingness. Finally, the sample included limited representation of remote rural and ethnic minority communities in Guangxi, where traditional gender norms are most persistent, potentially leading to an underestimation of stereotype intensity in these populations.

### 4.3. Unique contributions

This study makes 3 key unique contributions to the literature on gender and nursing in China. To the best of the authors’ knowledge, this work is among the limited studies that comprehensively explore the interplay of gender stereotypes, care satisfaction, and acceptance willingness across Guangdong and Guangxi, and it effectively captures regional heterogeneity in patient perceptions within these 2 culturally and socioeconomically distinct regions with diverse ethnic groups. Second, this study verifies that ethnic identity is not a dominant factor affecting patients’ acceptance of male interns in southern China, while clinical experience and overall satisfaction exert greater influence. This finding revises prior assumptions regarding the connection between ethnic cultural heritage and gender-related attitudes. Third, this research further promotes the contextual application of Social Role Theory and Gender Stereotype Theory in the Chinese setting. It confirms that regional socioeconomic and cultural factors can moderate the association between gender stereotypes and patient attitudes, thereby supplementing a macro-structural perspective to these frameworks that traditionally focus on individual-level analysis.

### 4.4. Practical implications and testable hypotheses

Based on correlational findings, we propose 2 testable hypotheses and targeted, concise practical recommendations for nursing education, clinical practice, and regional policy in Guangdong and Guangxi, with interventions tailored to inter-provincial differences (Guangxi’s stronger empathy stereotypes and lower satisfaction; Guangdong’s higher acceptance and open gender norms).

### 4.5. Testable hypotheses

Integrating culture-specific gender competence training into nursing curricula for male interns in Guangdong and Guangxi is associated with improved patient care satisfaction and a higher willingness to accept male interns among rural and ethnic minority patients.

Increasing male nurse representation in rural and ethnic minority healthcare settings in Guangxi is associated with reduced patient stereotypes of empathy as a feminine trait and higher acceptance of male nursing interns.

### 4.6. Core practical recommendations

Nursing education reform: Develop joint gender competence training modules for Guangdong and Guangxi nursing schools, integrating Lingnan and Zhuang cultural norms, empathy skill-building, and intimate care communication strategies. Establish a cross-provincial mentorship program pairing male interns with experienced male nurses from both provinces to share patient rapport-building practices for diverse cultural contexts.

#### 4.6.1. Clinical practice optimization

In Guangxi, expand male intern clinical exposure in rural and ethnic minority hospitals, with standardized care communication protocols aligned with local cultural norms to improve patient satisfaction. In both provinces, implement transparent patient communication about nursing intern teams and gender-neutral care competence, while providing flexible caregiver gender choices for intimate care to respect modesty norms without reinforcing default gender stereotypes.

#### 4.6.2. Cross-provincial policy collaboration

Establish a Guangdong-Guangxi male nursing workforce development mechanism, with Guangdong sharing its clinical training and patient acceptance improvement experiences to support Guangxi’s capacity building. Launch targeted public awareness campaigns in Guangxi highlighting the contributions of male nurses (including ethnic minority male nurses) to reduce empathy-related gender stereotypes, and incentivize male nurses to work in rural Guangxi to increase patient exposure to male caregivers.

## 5. Conclusion

This cross-sectional study identified key correlates of patients’ willingness to accept male nursing interns in Guangdong and Guangxi: care satisfaction, urban–rural background, and education level were positive predictors, while empathy-related gender stereotypes were the main negative predictor. Ethnic identity was unrelated to acceptance, with clinical care experiences exerting a more notable influence. The findings reflect the interaction between individual clinical interactions and regional socioeconomic-cultural factors, advancing the contextual application of gender theories in Chinese nursing research. Limitations include a cross-sectional design that cannot conduct causal inference and specialty-specific sampling, which restricts generalizability. Proposed testable hypotheses and targeted cross-provincial recommendations (education reform, clinical practice optimization, policy collaboration) lay a pragmatic foundation for subsequent intervention research. This study confirms that improving care quality and addressing structural barriers (e.g., rural-urban disparities in male nurse representation) are core to mitigating nursing gender stereotypes, and regionally tailored interventions are critical for culturally and socioeconomically diverse Chinese provinces.

## Author contributions

**Conceptualization:** Jun-Juan Liao, Xing-Xing Su, Xiao-Qin Qiu.

**Data curation:** Ming Zhou, Xing-Xing Su.

**Formal analysis:** Jun-Juan Liao, Xiao-Qin Qiu.

**Investigation:** Xing-Xing Su, Xiao-Qin Qiu.

**Methodology:** Jun-Juan Liao, Xiao-Lan Zeng, Xiao-Qin Qiu.

**Project administration:** Xiao-Qin Qiu.

**Resources:** Ming Zhou.

**Software:** Jun-Juan Liao, Xiao-Lan Zeng.

**Validation:** Ming Zhou.

**Visualization:** Ming Zhou.

**Writing – original draft:** Jun-Juan Liao, Xiao-Lan Zeng.
